# Do we need a scientific journal?

**DOI:** 10.47626/1679-4435-2021-194

**Published:** 2021-12-30

**Authors:** Francisco Cortes Fernandes

**Affiliations:** Scientific Director of the National Association of Occupational Medicine

With this question, I start a reflection on our Brazilian Journal of Occupational Medicine (*Revista Brasileira de Medicina do Trabalho*, RBMT). I believe that it is a timely reflection as we approach the publication of volume 20, in other words, RBMT has been published for approximately 20 years.

Returning to the initial question, the answer is obviously yes. However, I would like to add some arguments to this discussion.

We are well aware of the importance of disseminating scientific research in the society we live, fully permeated by science and technology. For these fields to develop, we need to disclose information. Therefore, we understand that scientific knowledge is part of society’s demands.

Nowadays, with the advent of the Internet and social media, access to information is becoming increasingly easier, but we need to be concerned with the reliability and quality of the information disclosed.

Some people believe that scientific research is disseminated only among scientists and academia. However, it is currently argued that knowledge must necessarily circulate through society, and, in this respect, it is essential that the traffic of information through the Internet and social media come from reliable sources.

Therefore, I understand that RBMT is fulfilling its role of disseminating occupational medicine knowledge in a consistent and easily accessible manner, with reliable and timely information regarding issues that arise in the daily practice of occupational physicians, as they are a result of observation of frontline health workers and professionals linked to academia.

And how did we start our journey?

In mid-2003, on the initiative of Professor René Mendes, the then president of the National Association of Occupational Medicine, RBMT was created. The journal had 80 pages containing an editorial, 6 original articles, 1 opinion article, and 1 case report. Among the authors, some have already left us, and others are active exponents of occupational medicine in Brazil.

The journal evolved until 2005, when it was faced with editorial continuity problems that persisted until 2010. Fortunately, the journal managed to keep its publication, although with a few pages.

In 2010, the journal’s publication frequency was resumed, with 2 annual issues and volumes of around 60 pages each.

In 2016, the frequency increased to 3 issues per year and, in 2017, we started the current format of 4 issues per year, which has been maintained since then, with an average of 132 pages per volume.

Therefore, we can state that our RBMT is consolidated and progressing to reach new horizons, compatible with the importance of our specialty, which, by the way, is the sixth specialty in number of specialists in Brazil.

In this trajectory toward adulthood, RBMT has published 426 articles as well as other types of publications, such as letters, case reports, opinion articles, systematic reviews, guidelines, book reviews, and brief communications, accounting for more than 4064 pages and 12,090 references.

The health professionals who most frequently publish their works in our RBMT, taking into account the principal investigator, are as follows: physicians, with 263 publications nurses, with 67 publications physical therapists, with 35 publications physical educators, with 23 publications psychologists, with 10 publications others, such as administrators, social workers, biologists, biochemists, dentists, social communicators, engineers, and physicists, with less than 4 publications each.

By distributing the publications across the specialty areas for the practice of occupational medicine, the following dispersion is obtained:

**Figure 1:**
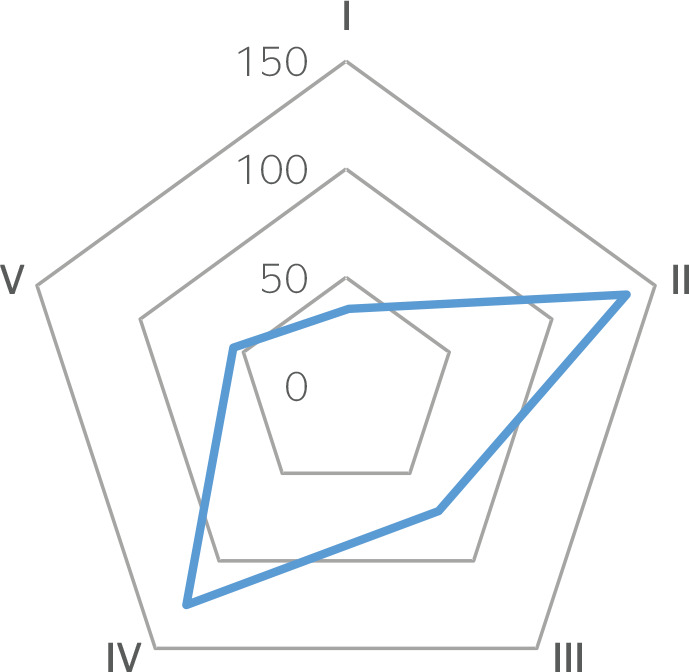
Dispersion of topics according to specialty area.

Thus, we can observe that comprehensive care to workers’ health and policies, health organization and management, safety, and environment are the most discussed topics in the published articles, demands that are undoubtedly important for the work of occupational physicians.

We have also had contributions from international institutions, such as from Italy, Mexico, Colombia, and Portugal, with almost 30 publications in our journal, which indicates a tendency toward internationalization.

It should also be noted the hard work of all RBMT editors and reviewers, who make the journal happen and assist in the writing of manuscripts submitted for publication.

Finally, I firmly believe that the journal will continue to drive occupational medicine knowledge and I call on all professionals interested in professional improvement to write and publish their experience, sharing knowledge and propelling our specialty to new heights, aiming at joint growth and focusing on promotion of workers’ health, the reason for our existence.

And let another 20 years come!

